# Transmission Dominance Under Random-Contact Intensification in Epidemic Networks: Multilayer Contact Network Simulation Study

**DOI:** 10.2196/90393

**Published:** 2026-05-20

**Authors:** Mingxuan Zhang, Tomohide Maekawa, Kaira Sekiguchi, Tadahiko Murata, Yukio Ohsawa

**Affiliations:** 1 Department of Systems Innovation, Graduate School of Engineering, The University of Tokyo, 7-3-1 Hongo, Bunkyo-ku, Tokyo 113-0033, Japan, Bunkyo-ku, Tokyo, 113-0033, Japan, 81 3-5841-6533; 2 Data Engineering, Trust Architecture Inc., Minato-ku, Tokyo, Japan; 3 Department of Systems Innovation, School of Engineering, The University of Tokyo, 7-3-1 Hongo, Bunkyo-ku, Tokyo, Japan, Bunkyo-ku, Tokyo, 113-8656, Japan; 4 D3 Center & Graduate School of Information Science and Technology, The University of Osaka, Ibaraki, Osaka, Japan

**Keywords:** digital epidemiology, contact networks, multilayer networks, epidemic modeling, SEIR, transmission heterogeneity, random contacts, public health informatics, agent-based simulation, COVID-19, Susceptible-Exposed-Infectious-Removed

## Abstract

**Background:**

In the context of COVID-19, infection spread through human contact networks remains a major public health challenge. Beyond cumulative infections and deaths, it is necessary to understand which contacts matter most, and which population segments contribute most to transmission under different social conditions. In multilayer urban networks with community structure, routine contacts coexist with incidental encounters, and it remains unclear whether incidental encounters can alter epidemic burden and the main contributors to transmission when per-layer contact caps and routine-contact minima are unchanged (for the nonrandom layers).

**Objective:**

Under explicit daily-contact constraints, we examined (1) how changing overall contact opportunities affects epidemic speed and burden when incidental encounters are held fixed, and (2) whether increasing incidental encounters alone, per-layer contact caps, and routine-contact minima fixed (for the nonrandom layers), shifts the main contributors to transmission from a high-contact group to a medium-contact group, and the underlying network mechanism.

**Methods:**

We constructed a multilayer potential contact network for a synthetic urban population of 10,038 individuals, representing household, school, workplace, distance-driven activities, and incidental encounters as separate layers. Daily contact networks were sampled from the potential network each day, and transmission was simulated for 120 days using a Susceptible-Exposed-Infectious-Removed model with vaccination. Individuals were classified into high-contact and medium-contact groups based on baseline contact intensity, and group contribution combined each group’s share of infectious individuals and its per-infectious effective transmission yield. Contact-constraint parameters were calibrated using an online survey in Tokyo and Kanagawa (n=1089), and scenario comparisons and parameter sweeps were used to locate the transition point.

**Results:**

With incidental encounters held fixed, higher overall contact opportunities produced earlier and higher epidemic peaks and larger cumulative infections and deaths, whereas reduced opportunities slowed and prolonged spread. Holding overall contact opportunities and routine contacts fixed, increasing incidental encounters shifted the main contributors to transmission: higher-contact individuals accounted for more effective transmissions at low incidental contact, whereas medium-contact individuals accounted for more beyond a clear transition point. Network visualization and schematics suggest a bridge-allocation mechanism, where stronger incidental contact adds cross-community bridges that more often terminate at medium-contact individuals and carry infection into less-affected communities. Across R=30 replicate runs under fixed settings, the dominance flip was consistently observed, and the estimated threshold W∗ showed a narrow but nonzero distribution (reported as median and IQR).

**Conclusions:**

In multilayer urban contact networks with community structure, our results indicate that intensifying incidental encounters can change the main contributors to transmission even when overall contact opportunities and routine contacts are unchanged. We present an analysis framework under explicit daily-contact constraints to identify this contributor shift and its transition point, supporting comparisons of intervention priorities across social contact conditions.

## Introduction

The spread of infection through human contact networks is a major public health challenge. Beyond predicting cumulative infections and deaths, authorities and practitioners need to understand which kinds of contacts matter most and which parts of the population contribute most to transmission under different social conditions. The COVID-19 pandemic has highlighted this need in urban settings where people combine stable routine contacts with diverse incidental encounters in workplaces, schools, leisure venues, and transport. Nonpharmaceutical interventions such as distancing, restrictions on gatherings, and changes in mobility patterns are all, implicitly or explicitly, attempts to reshape this network of contacts. Recent work has also proposed mobility-derived indices to quantify regional infection-expansion risk and connect movement heterogeneity to urban spreading dynamics in Tokyo [1].

Network-based epidemic models provide a natural language for these questions. In contrast to population-based compartmental models, which assume homogeneous mixing or a small number of contact groups, network approaches directly represent who can infect whom and how heterogeneous contact patterns shape spread [2-5]. A large body of work has examined how skewed degree distributions and scale-free–like structure affect epidemic thresholds and persistence [2,3], and how highly connected individuals or sets of nodes can disproportionately influence outbreak size and optimal targeting strategies [6-8]. These studies support the intuition that “high-contact” individuals tend to play an outsized role in transmission and are natural candidates for prioritized interventions.

At the same time, empirical and model-based studies have emphasized that real contact patterns are neither static nor single-layered. Urban contact structures arise from overlapping layers (households, workplaces, schools, leisure, and transport activities) and change over time as people move, schedule activities, and respond to policies [9-11]. Community structure further shapes epidemic dynamics—infections often grow first within dense modules and then jump across communities via a limited number of bridging contacts [12-15]. Temporal and multilayer network studies have shown that short-time reshuffling of contacts and interlayer bridges can strongly influence invasion routes and the timing of peaks [10,11,16]. Within this picture, a particularly important but hard-to-measure component is *incidental encounters*; random-like contacts outside one’s routine circle, such as chance meetings in restaurants, shops, or on public transport, which connect otherwise separated communities.

Existing work has clarified how network structure, temporal variability, and community organization affect epidemic thresholds and the overall success of interventions [3,5,11,14]. However, 2 related questions remain less explicit. First, while many studies recognize the importance of highly connected individuals or venues, fewer ask systematically *when* individuals with more moderate contact levels can become the main contributors to spread, especially in modular, multilayer settings. Second, many models implicitly bundle together 2 different aspects of contact behavior—the overall upper bound on how many different people one can meet in a day, and the narrower set of contacts that are deliberately maintained as routine contacts. From a policy and behavioral perspective, it is useful to distinguish between these 2 aspects and to ask how changes in random (incidental) encounters, over and above routine contacts, affect not only how many infections occur but also *who* drives transmission.

In this study, these questions are addressed by combining a constrained-contact framework, a multilayer urban contact network, and an extended Susceptible-Exposed-Infectious-Removed (SEIR) model. Building on earlier work on scale-free networks with behavioral limits [17], a setup is adopted that distinguishes a daily upper bound on distinct contacts from a required routine-contact minimum, and adds a separate control parameter for random (incidental) encounters. In line with earlier work on realistic urban contact networks and temporal contact structure [9,10], this framework is implemented on a multilayer contact network with community structure that represents households, workplaces, schools, distance-driven activities, and random contacts in a synthetic urban population representative of Tokyo and Kanagawa. On the resulting daily resampled contact networks, an extended SEIR model with vaccination and mild or severe branches is run [18], and the simulations are summarized in terms of how much high-contact and medium-contact individuals each contribute to transmission under different levels of random (incidental) encounters. Across these settings, it is found that increasing random (incidental) encounters can lead to a clear reversal in which group contributes more to spread; under low incidental-contact intensity, high-contact individuals are the main contributors, whereas under high incidental-contact intensity, individuals with medium contact levels embedded in multiple communities take over this role. This change is later formalized as a dominance flip between 2 degree-based groups, and the threshold at which it occurs is analyzed. Together with a structural interpretation in terms of community bridges, the framework offers a compact way to think about which contacts to reduce and which groups to prioritize when designing interventions in urban epidemics.

## Methods

### Notation and Key Quantities

We first summarize the core objects and quantities used throughout the paper.

#### Potential and Daily Contact Networks

Let $G^{\text{*}}\text{=}(V,E,l)$ denote the multilayer *potential* contact multigraph, where V is the set of individuals (agents), E is the set of potential undirected contacts, and l:E→L assigns each edge e∈E to a layer l(e∈L (household, school, workplace, distance-driven, random, etc). On each day t, a *daily contact graph* $G_{t}\text{=}(V,E_{t})$ is obtained by sampling a subset of edges $E_{t}\subseteq E$ from $G^{\text{*}}$ under the contact constraints defined below.

#### Contact constraints and the W–$m_{0}$ framework

We use 3 quantities to summarize daily contact opportunities:

- W: the per-layer daily *opportunity upper bound*, that is, the maximum number of distinct contacts an individual can realize within a given layer in a day.
- $m_{0}$: the per-layer *required routine-contact minimum*, that is, the minimum number of distinct routine contacts an individual must maintain within that layer for basic social functioning.
- $W_{random}$: the target mean potential random contacts per individual on the random layer (ie, the expected random-layer degree in the potential network G∗), implemented by choosing the Erdős–Rényi edge probability so that the expected random-layer degree is approximately $W_{random}$.

After required routine contacts are accounted for, any remaining daily contact opportunities within a layer can be allocated to incidental encounters. Under this multilayer implementation, the W–m_0 constraint is enforced within each layer rather than as a single cap on the cross-layer total. Following Ohsawa and Tsubokura [17], we refer to the pair $\left(W,m_{0}\right)$ as the W*–*$m_{0}$ *framework* for constrained social interaction.

#### Random Contacts and Incidental Encounters

In this paper, we use the term *random contacts* for close contacts that are not part of an individual’s deliberately maintained routine contacts. Behaviorally, these contacts correspond to the “chance encounters” captured in our survey questions on people met within 1.8  m for at least 5 minutes who were not planned in advance (eg, shop staff, other customers, and co-passengers in public transport). They are incidental in the sense that they arise from being present at shared venues rather than from prearranged meetings.

At the modeling level, random contacts are represented by a dedicated “random” layer in the potential network $G^{\text{*}}$. Potential edges on this layer are generated by an Erdős–Rényi mechanism, and the connection probability is chosen so that the expected degree on this layer is approximately $W_{random}$. Thus $W_{random}$ can be interpreted as the target mean number of such incidental encounters per individual on a representative day. In what follows, we use the phrases *random contacts* and *incidental encounters* interchangeably for this component.

#### Groups and Communities

**Figure 1.**
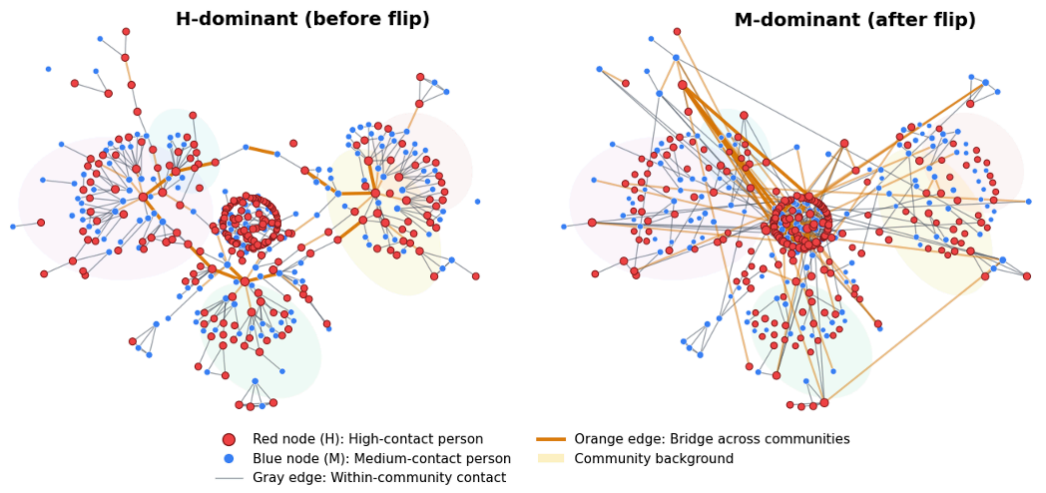
Same-node visualization before (left) and after (right) the flip. Red: high-contact, blue: medium-contact, orange: intercommunity bridges backbone (thicker=more important). H: high-contact; M: medium-contact.

We classify individuals into 2 degree-based groups on the potential network $G^{\text{*}}$. The high-contact group (H) contains the top 50% of nodes by degree, and the medium-contact group (M) contains the remaining 50%. When we refer to communities, we mean modules identified by a standard community-detection algorithm on an aggregated contact graph used for structural interpretation. These degree-based groups are distinct from communities; a community can contain both H and M individuals. For visualization in Figure 1, communities are computed on an aggregated reference graph; the details are provided in the Structural Interpretation of the H→M Dominance Flip subsection.

#### Dominance-Related Notation

Let i∈{H,M} index the high-contact group and medium-contact group (H/M groups). For a given contact-opportunity setting W, we define:

- $s_{i}(W)$: the share of infectious individuals belonging to group i;
- $n_{i}(W)$: the average number of effective transmissions generated by one infectious individual in group i;
- $e_{i}(W)\text{=}s_{i}(W)n_{i}(W)$: the *dominance score* of group i.

Intuitively, $e_{i}(W)$ measures how strongly infections in group i contribute to further spread at contact intensity W; if the number of infectious individuals were slightly increased in group i, the resulting increase in total infections would be larger for groups with larger $e_{i}(W)$ (ie, they have a stronger influence on spread). We say that group i *dominates* at W if $e_{i}(W)$ is the largest. The *dominance-flip threshold* $W^{\text{*}}$ is defined by, namely, the point at which the inequality $e_{H}\left(W\right)>e_{M}\left(W\right)$ for smaller W reverses to $e_{H}\left(W\right)<e_{M}\left(W\right)$ as W increases. In the simulations, we will evaluate these quantities as functions of the random-contact intensity $W_{random}$, using the notation above with W as the contact-opportunity argument.


$$W^{\text{*}}:e_{H}\left(W^{\text{*}}\right)\text{=}e_{M}\left(W^{\text{*}}\right)$$

### Model Overview

Our simulations combine a constrained, scale-free–like contact backbone with a multilayer structure and SEIR dynamics. We use a synthetic population (refer to Data and Synthetic Population subsection) to construct a multilayer potential contact network G* and define the H/M groups (refer to Multilayer Potential Contact Network and H/M Classification subsection). We then generate daily contact graphs Gt from G* under the W–m0 framework (refer to Daily Contact Sampling Under the W–$m_{0}$ Framework subsection) and simulate a discrete-time SEIR model with vaccination and mild or severe branches on these daily networks (refer to SEIR Dynamics With Vaccination subsection). Survey responses are used to calibrate baseline per-layer contact caps (refer to Survey-Based Calibration of Baseline Caps subsection). We report epidemic trajectories and dominance-based outcomes, including the dominance-flip threshold W* (refer to Outcome Measures and Analysis Plan, Baseline Epidemic Dynamics, Dominance Flip Between H and M groups, and Robustness Across Replicate Runs subsections).

Following Ohsawa and Tsubokura [17], we adapt a scale-free network with selfish spatiotemporal constraints as the backbone. In that construction, each node is subject to 2 constraints—an upper bound W on how many others it can meet and a lower cap $m_{0}$ on how many others it chooses to contact. In our multilayer implementation, these constraints are applied within each layer to capture distinct contact-generating mechanisms. Starting from multiple dense groups, nodes attach new edges while respecting these constraints, producing networks with pronounced communities and a limited number of nodes that bridge communities. In our setting, the nodes are individual agents in a nationally constructed synthetic population, and we embed this backbone into 10 layers (household, school, workplace, distance-driven, and random) that reflect the recorded attributes (refer to Data and Synthetic Population subsection).

Within this backbone, we adopt the W–$m_{0}$ framework from the study by Ohsawa and Tsubokura [17] to summarize daily contact opportunities at the layer level. W is interpreted as a per-layer daily opportunity upper bound on distinct contacts, and $m_{0}$ per-layer as a routine-contact minimum on deliberately maintained contacts. We further introduce a random-contact parameter $W_{random}$ that controls the expected number of additional random encounters per person by setting the Erdős–Rényi edge probability on the random layer so that the expected random-layer degree in the potential network $G^{\text{*}}$ is approximately $W_{random}$. In the experiments, we keep per-layer W and $m_{0}$ fixed and vary $W_{random}$ to represent different levels of random-contact intensity.

A central question in this paper is not only how many infections occur, but which group of individuals contributes most to the spread. To this end, for the high- and medium-contact groups H and $M\;defined\;on\;G^{*}$, we track two components: (1) the share of infectious individuals belonging to each group and (2) the average number of effective transmissions generated per infectious individual in that group. Their product denoted $e_{i}(W)$ in the Notation and Key Quantities subsection is served as a dominance score for group i∈{H,M}. As the random-contact parameter $W_{random}$ increases, the dominance scores for H and M can cross, yielding a *dominance flip* at a threshold $W^{\text{*}}$ where $e_{H}(W^{\text{*}})\text{=}e_{M}(W^{\text{*}})$. To quantify stochastic uncertainty under fixed parameter settings, we also conduct replicate simulations by varying the random seed and summarize the resulting variability of the estimated dominance-flip threshold $W^{\text{*}}$in the Robustness Across Replicate Runs subsection. The subsequent sections describe how we instantiate the data, networks, sampling scheme, and SEIR dynamics to study this flip.

### Data and Synthetic Population

We use a nationally constructed synthetic population and randomly sample 5000 households (10,038 individuals) from the Tokyo metropolitan area. The synthetic population is generated using a simulated-annealing–based synthetic reconstruction method without individual samples, which algorithmically assigns demographic and household attributes (eg, age, sex, and within-household kinship) to match available population statistics rather than representing real, identifiable individuals [19]. Although this subsample is small relative to the real population, such synthetic populations provide a standard way to reconstruct individual-level contact opportunities while preserving confidentiality [9,20].

**Figure 2.**
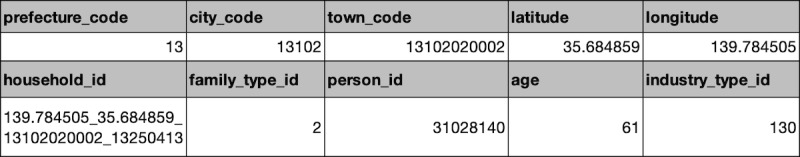
Example schema of individual-level fields used to generate potential contacts.

Each individual (agent) carries attributes that enable the reconstruction of urban contact layers. An example schema of these individual-level fields is shown in Figure 2. These attributes include:

- Geographic identifiers: *prefecture_code*, *city_code*, *town_code*, *latitude*, and *longitude*.
- Household and personal identifiers: *household_id*, *person_id*, *family_type_id*, and *age*.
- Organizational attributes: for example, *industry_type_id*; when available, *school_id*, and *school_grade*.

These attributes support the construction of a multilayer potential contact network on the common vertex set, as described in the Multilayer Potential Contact Network and H/M Classification subsection. In particular, household, school, and workplace layers, together with age-stratified mixing, are chosen to be consistent with empirical contact matrices and their cross-country projections [21,22].

### Multilayer Potential Contact Network and H/M Classification

#### Overview

On the common vertex set V introduced in the Notation and Key Quantities subsection, we construct a multilayer *potential* contact multigraph $G^{\text{*}}\text{=}(V,E,l)$. Each node v∈V represents an individual (agent), each edge e=(u,v)∈E represents a potential undirected contact between 2 individuals u and v, and l:E→L assigns each edge to a layer l(e∈L.) The 10 layers and their generation rules are summarized in Table 1.

**Table 1.** Layer definitions and generation rules.

Layer	Rule or description
Strongly coupled layers	
household	Fully connected within each household.
school_classmate	Densely connected among classmates in the same town and grade.
industry_colleague	Dense local clusters within the same industry segment.
best_friends	Small friendship groups in which most pairs are connected.
Organizational layers	
industry	Workplace organization; contacts are counted toward the routine-contact minimum $m_{0}$.
school	School organization; contacts are counted toward the routine-contact minimum $m_{0}$.
Distance-driven layers	
dining_out	Dining-out; edge probability decays with geographic distance [16,23].
amusement	Leisure or recreation; distance decay [16,23].
routine	Commuting or errands; distance decay [16,23].
Random layer	
random	Chance encounters (random contacts) represented by an Erdős–Rényi layer. For each potential random edge, the connection probability is chosen so that the expected random-layer degree matches the target $W_{random}$ [24]. We use an Erdős–Rényi (ER) generator for the random layer as a minimal and controllable baseline for incidental mixing. This choice allows us to isolate the effect of increasing random encounters ( $W_{random}$) without introducing additional structural assumptions (eg, hub- or venue-specific heterogeneity) that would complicate interpretation of the dominance flip.

#### H/M Classification

To later compare which type of individuals dominates spread, we partition nodes into 2 degree-based classes on the potential network $G^{\text{*}}$. The H (high-contact) class consists of the top 50% of nodes by degree (upper quantile q=0.5), and the M (medium-contact) class consists of the remaining 50%. This is a degree-based *classification*, not a ranking of importance; it separates nodes into 2 contact-intensity classes, following standard practice in heterogeneous network analyses where early spread and thresholds are shaped by degree heterogeneity [3,21].

### Daily Contact Sampling Under the W–$m_{0}$ Framework

The potential network $G^{\text{*}}$ encodes *where* contacts can occur under the per-layer caps $\left(W,m_{0},W_{random}\right)$ defined in the Notation and Key Quantities subsection. On each day t, we generate a *daily contact graph* $G_{t}\text{=}(V,E_{t})$ by sampling a subset of edges $E_{t}\subseteq E$ from $G^{\text{*}}$. Sampling is carried out for each layer using layer-specific probabilities (Table 2), and all layers are sampled within the same daily step.

**Table 2.** Layer-wise daily sampling probabilities $p_{l}$.

Layer	$p_{l}$Probability ()
household	1.00
industry_colleague	1.00
industry	0.20
school_classmate	1.00
school	0.20
best_friends	0.10
dining_out	0.10
amusement	0.10
routine	0.10
random	0.10

### Layer-Wise Sampling

For each layer l∈L, each potential edge in that layer is active on day t with probability $p_{l}$ and inactive with probability $1\text{-}p_{l}$, independently across edges and days. The 4 groups of layers in Table 1 share the same sampling mechanism but use different probabilities $p_{l}$.

Strongly coupled layers (A)—household, school_classmate, industry_colleague, and best_friends—represent stable, repeated contacts. Their sampling probabilities are set high (Table 2), so that most potential contacts in these layers appear on a typical day.

Organizational layers (B)—industry and school—represent routine organizational contacts such as work and class attendance. Edges in these layers are constructed as maintainable contacts to meet the per-layer routine-contact minimum $m_{0}$ in the potential network $G^{\text{*}}$ and are then activated independently day by day with probabilities $p_{l}\text{=}0.20$. Distance-driven layers (C)—dining_out, amusement, and routine—are constructed with edge probabilities that decay with geographic distance [16,23] and sampled with $p_{l}\text{=}0.10$. Finally, random-layer edges (D) represent chance encounters. Their potential structure is determined by the random-contact parameter $W_{random}$ when $G^{\text{*}}$ is built, and on each day, we activate each potential random edge independently with probability $p_{l}\text{=}0.10$. Therefore, the expected realized random-layer degree per day is approximately P_random_×W_random_. Because this sampling is repeated independently for each day, the sequence of daily graphs $\left\{G_{t}\right\}$ exhibits natural day-to-day variability even when the underlying potential network $G^{\text{*}}$ is fixed.

### SEIR Dynamics With Vaccination

#### Overview

**Figure 3.**
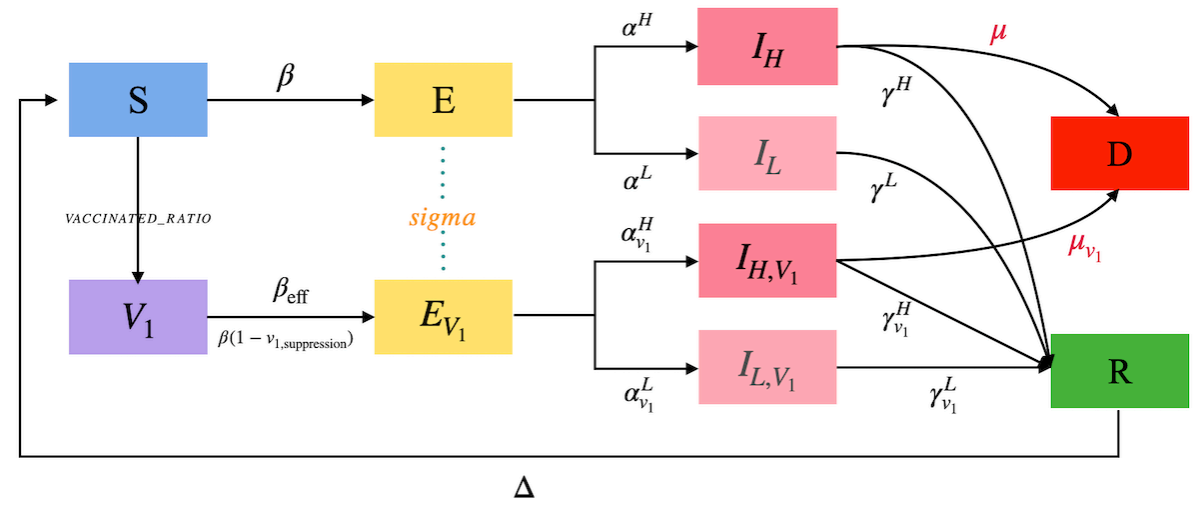
Extended Susceptible-Exposed-Infectious-Removed schema with vaccination and mild or severe branches. Arrows indicate possible daily transitions on the contact network.

The epidemic dynamics are modeled by a discrete-time SEIR framework that operates on the daily contact networks $G_{t}$ described in the Daily Contact Sampling Under the W–$m_{0}$Framework subsection. Our agent-based implementation follows established individual-level designs for city-scale scenarios [5,25]. Each simulation day corresponds to one time step (Δ=1d), during which the daily contact network is sampled, infection events are evaluated along its edges, and compartmental states are updated synchronously. Synchronous updates mitigate order effects in network transmission and align with standard practice in discrete-time epidemic modeling [26]. The model extends the standard SEIR structure by introducing a mild or severe branching and a single-dose vaccination state to capture heterogeneity in disease progression (Figure 3); vaccine-effect modeling follows standard field-study formalizations [27] and network-epidemic treatments [5].

#### Model Structure

Each agent exists in one of the following compartments: susceptible (S), vaccinated susceptible ($V_{1}$), exposed (E), vaccinated exposed ($E_{V_{1}}$), infectious–mild ($I_{L}$), infectious–severe ($I_{H}$), vaccinated infectious–mild ($I_{L,V_{1}}$), vaccinated infectious–severe ($I_{H,V_{1}}$), recovered (R), and deceased (D). Transitions between these states are governed by fixed parameters, such as infection probabilities, progression rates, and mortality rates, summarized in Table 3.

**Table 3.** Key epidemiological parameters (baseline values) and sources. All epidemiological parameters in Table 3 are applied uniformly across layers; layer differences enter through the contact-network construction and daily sampling scheme (Table 2).

Parameter (symbol)	Value	Sources
Per-contact infection probability (β)	0.25	Kuniya [28] and Liu et al [29]
Vaccine infection suppression (v_1_; relative susceptibility, $v_{1}$)	0.60	Arashiro et al [30] and Fowlkes et al [31]
Severe fraction, unvaccinated ($\alpha_{H}$)	0.18	Matsunaga et al [32] and The Novel Coronavirus Pneumonia Emergency Response Epidemiology Team [33]
Severe fraction, vaccinated ($\alpha_{H,V1}$)	0.04	Arashiro et al [30] and Birhane et al [34]
Recovery rate, severe ($\gamma_{H}$; day^−1^	0.07	Matsunaga et al [32], report by World Health Organization [35], and Ohbe et al [36]
Recovery rate, mild ($\gamma_{L}$; day^−1^)	0.20	Matsunaga et al [32] and report by World Health Organization [35]
Mortality, severe unvaccinated (μ)	0.20	Matsunaga et al [32], report by World Health Organization [35], and Ohbe et al [36]
Mortality, severe vaccinated ($\mu_{V1}$)	0.03	Arashiro et al [30] and Birhane et al [34]
Vaccinated ratio at t=0	0.70	Assumed baseline vaccination coverage informed by reported vaccination coverage in Tokyo, Japan

#### Daily Transmission Dynamics on $G_{t}$

For each day t, infections are evaluated along the edges of the daily contact graph $G_{t}$. Every infectious individual ($I_{H}$, $I_{L}$, $I_{H,V_{1}}$, and $I_{L,V_{1}}$) attempts to infect susceptible or vaccinated neighbors. Each infectious contact involving an individual in state S leads to infection with probability β, and each infectious contact involving an individual in state $V_{1}$ leads to infection with probability $\beta_{V_{1}}\text{=}v_{1}\beta$. Infection events are drawn independently across edges.

Conditioned on the new infections on day t, compartmental transitions are then applied synchronously; newly infected individuals move from S or $V_{1}$ to E or $E_{V_{1}}$, exposed individuals progress to $I_{L}$ or $I_{H}$ (and to $I_{L,V_{1}}$ or $I_{H,V_{1}}$ in the vaccinated branch), and infectious individuals either recover (R) or die (D) according to the rates in Table 3. Thus, each simulation day consists of (1) sampling a daily contact graph $G_{t}$ from the potential network $G^{\text{*}}$ (refer to Daily Contact Sampling Under the W–$m_{0}$ Framework subsection), (2) propagating infection along the edges of $G_{t}$, and (3) updating epidemiological states in discrete time.

The number of potential infectious contacts per day is determined by the realized daily network $G_{t}$, which in turn depends on the contact constraints $\left(W,m_{0},W_{random}\right)$ and the layer-wise sampling probabilities $p_{l}$ defined in the Notation and Key Quantities, Model Overview, Data and Synthetic Population, Multilayer Potential Contact Network and H/M Classification, and Daily Contact Sampling Under the W–$m_{0}$ Framework subsections. In particular, changing $W_{random}$ modifies the intensity of chance encounters on the random layer while keeping the construction of other layers fixed.

### Simulation Setup and Calibration

#### Scenario Design Under the W–$m_{0}$ Framework

The main simulations were run for T=120 days. Unless noted otherwise, the random-contact parameter was fixed at $W_{random}\text{=}5$, and 3 overall contact-intensity conditions were compared under the W–$m_{0}$ framework defined in the Notation and Key Quantities subsection: (1) a twofold-intensity setting (Attr0, W×2), (2) a baseline setting (Attr1), and (3) a half-intensity setting (Attr2, W/2). In each setting, the per-layer opportunity caps W and routine-contact minimum $m_{0}$ follow the calibration described below, with $m_{0}$ held fixed across the 3 scenarios. Layer-wise parameter values are summarized in Table 4.

**Table 4.** Layer-wise scenario settings for Attr0 (W×2), Attr1 (baseline), and Attr2 (W/2). $m_{0}$ held constant.

Layer	Attr0 (*W*^a^×2)	Attr1 (baseline)	Attr2 (*W*/2)
Household	Fully connected within household	Fully connected within household	Fully connected within household
Workplace (*m*_0_^b^)	Fully connected within workplace (average size≈3)	Fully connected within workplace (average size≈3)	Fully connected within workplace (average size≈3)
School ($m_{0}$)	Fully connected within same city and age cohort (≤15)	Fully connected within same city and age cohort (≤15)	Fully connected within same city and age cohort (≤15)
Workplace ($W,m_{0}$)	$W\text{=}6;m_{0}\text{=}3$	$W\text{=}5;m_{0}\text{=}3$	$W\text{=}4;m_{0}\text{=}3$
School ($W,m_{0}$)	$W\text{=}10;m_{0}\text{=}4$	$W\text{=}7;m_{0}\text{=}4$	$W\text{=}5;m_{0}\text{=}4$
Dining-out	*W*=25; *m*_0_=5; *ε*^c^=0.01	W=15; $m_{0}\text{=}5;\varepsilon \text{=}0.01$	W=6; $m_{0}\text{=}5;\varepsilon \text{=}0.01$
Leisure or amusement	W=60; $m_{0}\text{=}5;\varepsilon \text{=}0.50$	W=30; $m_{0}\text{=}5;\varepsilon \text{=}0.50$	W=15; $m_{0}\text{=}5;\varepsilon \text{=}0.50$
Shopping or errands	W=30; $m_{0}\text{=}5;$ ε=0.0001	W=15; $m_{0}\text{=}5;$ ε=0.0001	W=7; $m_{0}\text{=}5;$ ε=0.0001
Random	ER^d^ sampling with *P*rand(W_random_); expected random-layer degree calibrated to W_random_	ER sampling with *P*rand(*W*_random_); expected random-layer degree calibrated to *W*_random_	ER sampling with *P*rand(*W*_random_); expected random-layer degree calibrated to *W*_random_

a *W*: per-layer opportunity cap.

b *m*_0_: routine-contact minimum.

c distance-decay strength (larger⇒stronger locality).

d ER: Erdős–Rényi.

#### Survey-Based Calibration of Baseline Caps

The calibration relied on a 2-stage online survey consisting of a screening module and a main diary module. In the screening survey (July 7‐13, 2023), 639,723 invitations were distributed, and 14,975 responses were received; 1920 respondents provided consent. For the main diary survey (July 20‐26, 2023), 1700 invitations were distributed to the same panel, and 1438 complete submissions were received. After quality-control checks, 349 responses were excluded, resulting in a final analytic sample of 1089 respondents.

Using the main diary survey, we calibrated the baseline contact parameters for Attr1 from self-reported daily activities and close contacts. The survey was conducted between July 20 and July 26, 2023, among residents of Tokyo and Kanagawa prefectures. Eligible respondents were men and women aged 20‐69 years living in these prefectures. The screening and diary modules were administered to the same registered online panel; responses flagged by basic quality-control checks were excluded, as summarized above.

For each respondent, the questionnaire first asked about basic attributes and planned outings over a 5-day window (July 20-24, 2023). Respondents were then asked to select the single day within this window on which their outings and movements were the most frequent. They first confirmed whether the planned outings actually occurred within the window and then selected the busiest day from those realized days. Detailed questions on places, timing, duration, and contacts were asked only for this selected day to standardize reporting and reduce recall burden. For this “busiest day,” the survey recorded which types of places were visited (school, workplace, restaurants, leisure facilities, shopping venues, public transport, etc), at what times of day, and for how long. Reported durations for place visits did not include travel time between locations. Travel time was recorded under public transport. These reports were grouped into the layers used in our model (household, workplace, school, dining-out, leisure, shopping or errands, and transport), and the mean number of outings per layer on the busiest day was used to set the baseline per-layer opportunity caps W in Table 4. All survey summaries were computed as unweighted arithmetic means across respondents.

The survey also collected information on close contacts on the same day. For each outing type, respondents reported how many people they met at a distance of less than 1.8 m for at least 5 minutes. Contact counts were reported as cumulative numbers of distinct persons. Companions who went out together with the respondent were not included from the close-contact counts. Respondents were further asked how many of these contacts were with people they had planned to meet (eg, colleagues, classmates, family members, and friends) and how many were people they encountered by chance (eg, shop staff and other customers). Chance encounters were defined as close contacts not planned in advance, including strangers in the setting (eg, staff or other customers) and unplanned encounters with acquaintances. The mean number of planned close contacts per layer was used as a routine-contact minimum estimate and mapped to the baseline values of $m_{0}$ in Table 4. The mean number of chance encounters across relevant settings (eg, restaurants, leisure facilities, shopping, and transport) was used to determine a plausible range for the random-contact parameter $W_{random}$; within this range, we selected $W_{random}\text{=}5$ as the baseline. The twofold-intensity (Attr0) and half-intensity (Attr2) conditions were then obtained by multiplying or halving the baseline opportunity caps W for each layer, while keeping $m_{0}$ fixed. Therefore, the survey outputs were used to calibrate baseline opportunity and routine-contact caps in the model, rather than to estimate population-level contact rates. Ethics approval and related procedures are described in the next Ethical Considerations subsection.

#### Outcome Measures and Analysis Plan

**Figure 4.**
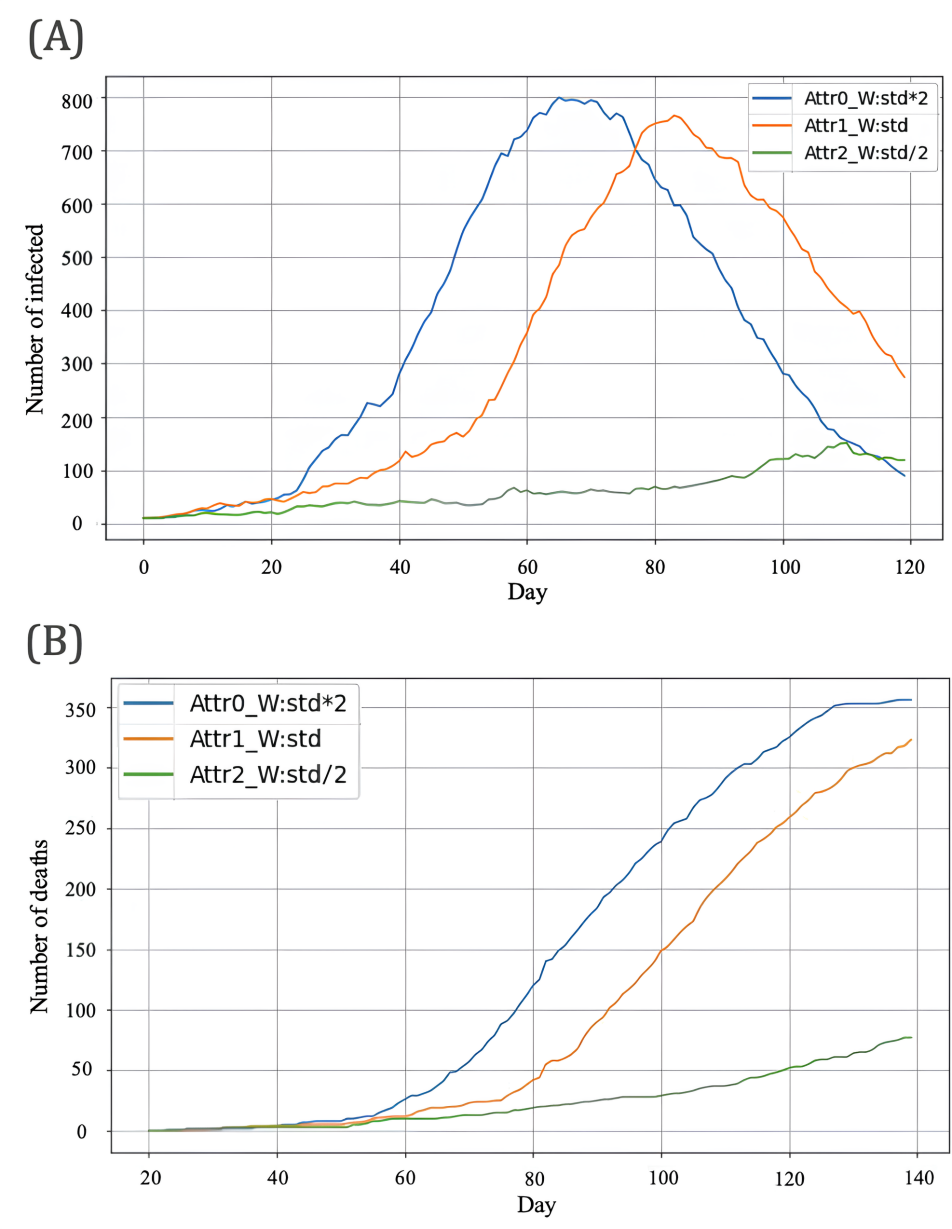
Outcomes under 3 contact-intensity scenarios ($w_{random}=5$). (A) Infections and (B) cumulative deaths.

**Figure 5.**
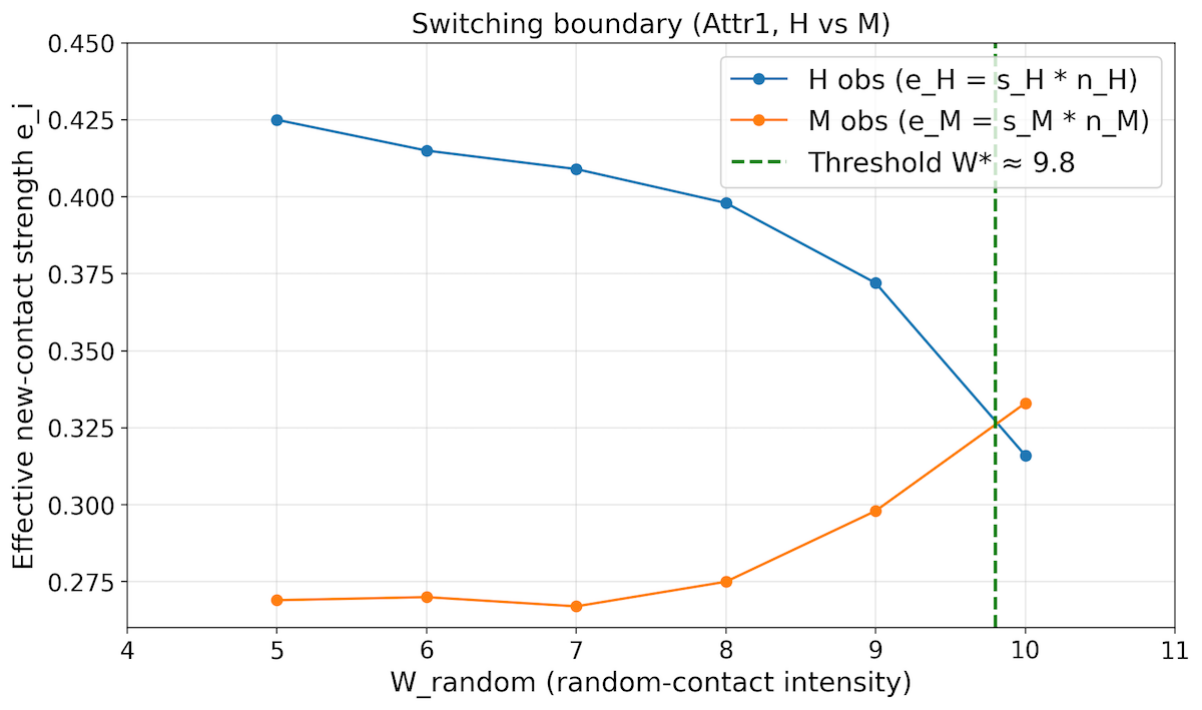
Difference in dominance scores between the high-contact group (H) and medium-contact group (M) under the baseline (Attr1) condition. The horizontal axis is parameterized by the random-contact intensity $w_{random}$. H: high-contact; M: medium-contact.

**Figure 6.**
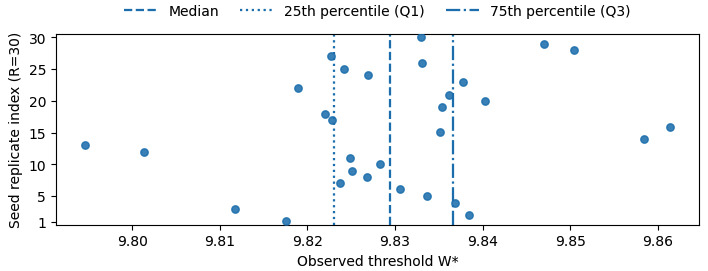
Distribution of the estimated dominance-flip threshold W* across replicate runs (boxplot with individual replicate estimates).

We report (1) epidemic trajectories (infections and cumulative deaths; Figure 4) and (2) dominance-related measures for the 2 degree-based groups H and M defined on the potential network (G*; the Notation and Key Quantities subsection). For each setting, we compute the dominance scores ($e_{i}(W)\text{=}s_{i}(W)n_{i}(W)$) and summarize dominance using ($e_{H}(W)\text{=}e_{M}(W)$; Figure 5). The dominance-flip threshold ($W^{\text{*}}$) is defined as the crossing point where $e_{H}(W^{\text{*}})\text{=}e_{M}(W^{\text{*}})$ (refer to Notation and Key Quantities subsection). To quantify uncertainty under fixed settings, we report the replicate distribution of ($W^{\text{*}}$; median and IQR) and the fraction of replicates with a crossing within the tested grid ($W_{random}$ in studies by Kiss et al [5] and Holme and Saramäki [10]; Figure 6).

We compare the 3 scenario settings (Attr0, Attr1, and Attr2) under the calibrated layer-wise caps in Table 4 with ($W_{random}$=5) unless noted (refer to Baseline Epidemic Dynamics subsection). To identify the dominance flip, we hold baseline layer parameters fixed (Attr1) and vary ($W_{random}$) from 5 to 10; for each value, we estimate ($e_{H}(W$)) and ($e_{M}(W)$) from simulated trajectories and estimate ($W^{\text{*}}$) from their crossing (refer to Dominance Flip Between H and M Groups subsection). We then repeat the threshold estimation across replicate runs with different random seeds and summarize the variability of ($W^{\text{*}}$; refer to Robustness Across Replicate Runs subsection).

### Ethical Considerations

#### Ethics Approval

This study used two types of data: (1) a synthetic population dataset and (2) an anonymized secondary dataset derived from a 2-stage online survey administered via an internet research company’s registered panel to residents of Tokyo and Kanagawa on July 20-July 26, 2023, used only for model calibration. The survey study and its secondary use were reviewed and approved by the Research Ethics Committee of the School of Engineering, The University of Tokyo (reference KE22-12). All data collection and secondary use were conducted within the approved scope. The synthetic population dataset does not contain information that can identify real individuals and was used only for simulation analyses; therefore, no additional ethics review was required for analyses based solely on the synthetic dataset.

#### Informed Consent

Informed consent procedures were implemented in the original survey study under the approved protocol (KE22-12). This work did not recruit participants and used only anonymized secondary survey data for calibration.

#### Privacy and Confidentiality

This study did not collect any personally identifiable information. The survey-derived dataset was provided and analyzed in an anonymized form, and results are reported only as aggregate summaries used to set baseline opportunity and routine-contact caps. The synthetic population dataset does not include identifiers of real individuals. Data were stored and analyzed on access-restricted systems.

#### Participant Compensation

Participant compensation, if any, was handled in the original survey study as specified and approved under the ethics protocol (KE22-12). No compensation was provided for the present simulation study.

## Results

### Overview

Using the methodology in the Methods section and the simulation setup and calibration described in the Simulation Setup and Calibration subsection, we examine how changes in contact caps affect epidemic dynamics and the dominance measure $e_{i}(W)$ for the high- and medium-contact groups, H and M defined in the Notation and Key Quantities subsection. The Baseline Epidemic Dynamics subsection presents baseline epidemic curves under the calibrated contact constraints. The Dominance Flip Between H and M Groups subsection then analyzes how the dominance scores $e_{H}(W)$ and $e_{M}(W)$ change as random-contact intensity varies and identifies a dominance-flip threshold. The Robustness Across Replicate Runs subsection evaluates robustness across replicate runs, and the Structural Interpretation of the H→M Dominance Flip subsection interprets this flip in terms of network structure on the multilayer backbone.

### Baseline Epidemic Dynamics

Under the baseline random-contact setting ($W_{random}\text{=}5$), the simulated epidemic curves exhibited typical SEIR-type dynamics (Figure 4). Higher overall contact opportunities (W×2, Attr0) produced an earlier and higher infection peak as well as a larger cumulative number of deaths, whereas the half-intensity condition (W/2, Attr2) resulted in slower and more prolonged spread. A more detailed interpretation of how overall contact opportunities affect epidemic speed and size is deferred to the Discussion section.

### Dominance Flip Between H and M Groups

We next examine how random-contact intensity affects which group of individuals dominates the spread. Throughout this subsection, all layer parameters are fixed to the baseline (Attr1) values in Table 4, and we vary only the random-contact intensity $W_{random}$ while holding the per-layer opportunity upper bound W and the per-layer routine-contact minimum $m_{0}$ fixed (for the nonrandom layers).

As defined in the Notation and Key Quantities subsection, the dominance score for group i∈{H,M} at a given contact-opportunity setting W is


$$e_{i}\left(W\right)\text{=}s_{i}\left(W\right)n_{i}\left(W\right)$$

where $s_{i}(W)$ is the share of infectious individuals belonging to group i and $n_{i}(W)$ is the average number of effective transmissions generated by one infectious individual in that group. In the simulations below, we evaluate $e_{i}(W)$ at different values of the random-contact intensity $W_{random}$, using W as the contact-opportunity argument.

For each tested value of $W_{random}$ between 5 and 10, we run multiple epidemics under the baseline (Attr1) setting and compute the dominance scores $e_{H}(W)$ and $e_{M}(W)$ from the simulated trajectories. We then fit smooth curves to their dependence on random-contact intensity. A dominance flip occurs when the dominant group changes between H and M as W varies (refer to Notation and Key Quantities subsection). Under the baseline condition, the fitted curves cross near W*≈9.82.

As we will next show in the Robustness Across Replicate Runs subsection, repeated runs yield a narrow but nonzero range for this estimate (median 9.8294, IQR 9.8231‐9.8367), so we report W* as an estimated range within this setting rather than a fixed constant. For example, when $W_{random}\text{=}9$, the dominance scores are approximately $e_{H}\approx 0.37$ and $e_{M}\approx 0.30$, so the high-contact group H still dominates. When $W_{random}\text{=}10$, they become $e_{H}\approx 0.32$ and $e_{M}\approx 0.33$, so the medium-contact group M becomes dominant. These values illustrate the transition from an H-dominant regime to an M-dominant regime as random-contact intensity increases.

Figure 5 summarizes this pattern through the difference $e_{H}(W)\text{-}e_{M}(W)$. The horizontal axis is parameterized by the random-contact intensity $W_{random}$, and the sign of $e_{H}(W)\text{-}e_{M}(W)$ changes at the threshold $W^{\text{*}}\approx 9.82$; it is positive for $W_{random}≲9.8$ (H-dominant) and negative for $W_{random}≳9.8$ (M-dominant).

### Robustness Across Replicate Runs

The daily contact sampling and epidemic progression in our simulations are stochastic. To assess how this stochasticity affects the estimated dominance-flip threshold, we conducted replicate simulations under identical model parameters while varying the random seed. In each replicate, we estimated the dominance-flip threshold W* using the same definition and estimation procedure as in the Notation and Key Quantities and Dominance Flip Between H and M Groups subsections, based on the crossing point of the dominance scores $e_{i}(W)\text{=}s_{i}(W)n_{i}(W)$.

Across replicates, the estimated W* values were tightly concentrated (Figure 6). The median estimated threshold was 9.8294 (IQR 9.8231-9.8367). Using the same tested grid of the random-contact intensity parameter ($W_{random}$∈ [5,10]), a dominance-score crossing within this range was observed in 30 out of 30 replicates. These results indicate that the dominance flip is consistently observed under the fixed parameter setting, and that the estimated threshold has a measurable uncertainty range.

### Structural Interpretation of the H→M Dominance Flip

The previous subsection showed that, under the W–$m_{0}$ framework, the dominance score $e_{i}(W)$ for the high-contact group H and the medium-contact group M crosses at a threshold $W^{\text{*}}\approx 9.8$ in terms of the random-contact parameter $W_{random}$. We now examine how the multilayer structure of the network changes around this threshold. We use a same-node visualization of a representative subgraph to compare the allocation of intercommunity bridges before and after the flip. This same-node visualization is intended as a mechanistic illustration rather than as an estimator of W*; robustness of the flip threshold is assessed separately through replicate runs.

### Same-Node Visualization

From the realized daily contact graphs on a fixed simulation day, we extract a same-node subgraph (Figure 1) anchored on high-contact individuals. We first identify the 3 highest-degree nodes in group H, take their 1‐2 hop neighborhoods, and cap the size at about 250 nodes. To provide a stable reference for both panels, we detect communities (modules) on the aggregated edge union graph of the 2 panels (same node set) using a standard label-propagation algorithm, yielding a nonoverlapping partition that is used only for visualization and for defining cross-community edges. An *intercommunity bridge* is an edge whose end points belong to 2 different communities. Among these cross-community edges, we highlight a bridge backbone ranked by edge betweenness centrality; higher-ranked edges are drawn thicker and in orange. Red nodes indicate high-contact individuals (H), and blue nodes indicate medium-contact individuals (M).

The left panel in Figure 1 corresponds to a setting with weaker random contact ($W_{random}\text{§amp;lt;}W^{\text{*}}$), where H dominates. Here, most of the thick orange bridges connect adjacent background regions and have at least 1 red end point; many visually prominent bridges start from the central red core and end at red nodes in nearby communities. Blue nodes appear on bridges mainly as peripheral end points at the edges of communities. The right panel corresponds to stronger random contact ($W_{random}\text{§amp;gt;}W^{\text{*}}$), where M dominates. In this case, orange bridges are more numerous and many of the thick orange edges run across nonadjacent background regions, linking visibly distant parts of the layout. Compared with the left panel, a larger fraction of these thick bridges now have blue end points, and several orange edges connect 2 blue nodes. Thus, medium-contact individuals more often sit at the ends of intercommunity bridges and connect a larger number of distinct communities. This reallocation of bridges increases the effective reach of the groups M and raises $e_{M}(W)$ relative to $e_{H}(W)$, consistent with the dominance flip observed in the Dominance Flip Between H and M Groups subsection.

## Discussion

### Principal Findings

Within the multilayer, daily-resampled contact network and discrete-time SEIR model described in the Methods and Results sections, we examined how overall contact opportunities and random-contact intensity shape epidemic outcomes and the leading contributors to spread. First, under fixed random-contact intensity ($W_{random}\text{=}5$), increasing the opportunity upper bound W (twofold vs baseline vs half; Figure 4) produced earlier and higher peaks and larger cumulative deaths. Thus, within the calibrated range of parameters, higher overall contact opportunities directly increased both the speed and scale of epidemic expansion. Second, focusing on the standard overall-intensity condition and varying the random-contact parameter $W_{random}$, we observed a *dominance flip* between high-contact (H) and medium-contact (M) individuals: for low $W_{random}$ the H group dominates, whereas for high $W_{random}$ the M group dominates (refer to Dominance Flip Between H and M Groups subsection; Figure 5). Dominance is measured by the score $e_{i}(W)\text{=}s_{i}(W)n_{i}(W)$ defined in the Notation and Key Quantities subsection, and the empirical threshold at which $e_{H}(W)\text{=}e_{M}(W)$ occurs is $W_{random}^{\text{*}}\approx 9.8$. These results show that changes in random-contact intensity can reshuffle which part of the population contributes most to transmission, even when overall contact opportunities are held fixed.

### Mechanistic Interpretation

The dominance flip arises from the interaction of 2 effects, which we summarize here and illustrate using 2 complementary views (Figures1  7).

**Figure 7.**
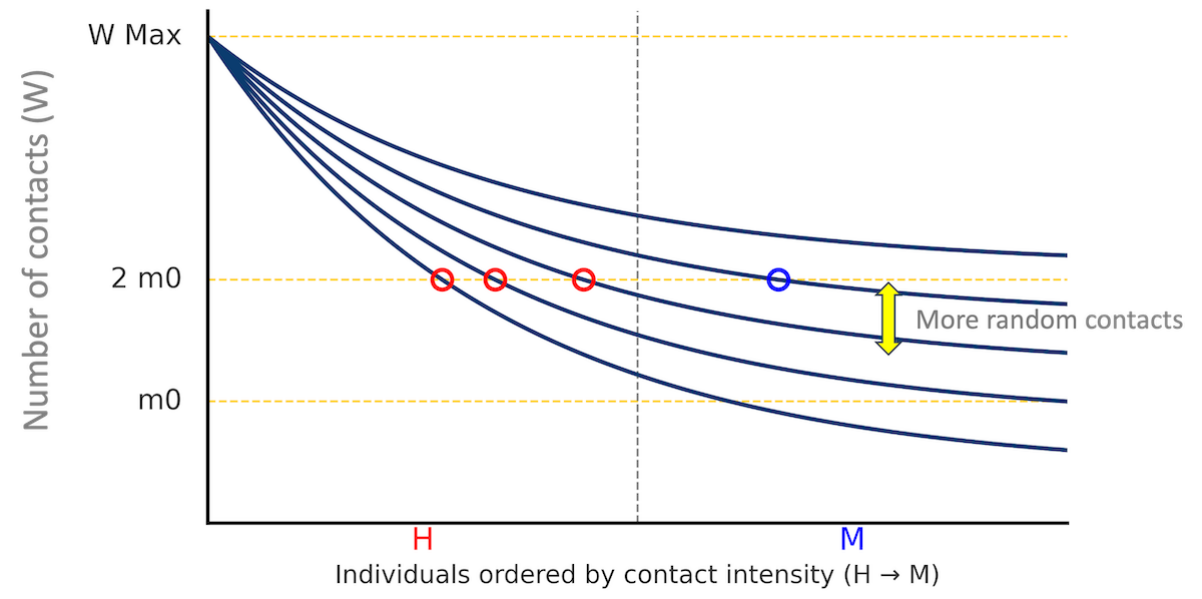
Schematic: individuals ordered by contact intensity (H→M, horizontal); daily contacts W (vertical). Stronger random contact shifts the curves upward. Dashed lines: $m_{0}$ and $2m_{0}$. H: high-contact; M: medium-contact.

- *Saturation on the H side*: In the calibrated networks, H nodes already maintain many contacts to meet the routine-contact minimums $\left(W,m_{0}\right)$. When random contact is increased by raising $W_{random}$ while keeping the per-layer caps W and $m_{0}$ fixed (for the nonrandom layers), the number of *new* partners that H nodes can add is limited. As a result, the effective transmission rate $n_{H}(W)$ increases only modestly.
- *Bridges reallocating toward M*: Higher random contact generates more intercommunity bridges and longer-range connections. As $W_{random}$ increases, a larger fraction of these bridges terminate at M nodes rather than H nodes (Figure 1, right). This raises the effective transmission rate $n_{M}(W)$, and with group sizes $s_{i}(W)$ changing slowly, the dominance score $e_{M}(W)\text{=}s_{M}(W)n_{M}(W)$ eventually exceeds $e_{H}(W)$.

To provide an intuitive interpretation within the same contact-constraint framework, Figure 7 illustrates a schematic to meet the routine-contact minimums $\left(W,m_{0}\right)$. Individuals are ordered on the horizontal axis by baseline contact intensity on the potential network $G^{\text{*}}$ (left: higher-degree nodes, mainly H; right: lower-degree nodes, mainly M), and the vertical axis represents expected daily contacts. As random-contact intensity $W_{random}$ increases while W and $m_{0}$ are held fixed, the curves shift upward. Following Ohsawa and Tsubokura [17], the dashed lines mark $m_{0}$ and $2m_{0}$ as reference levels. In this schematic, increasing $W_{random}$ raises contacts more on the M side where residual opportunity under $\left(W,m_{0}\right)$ remains, so more M nodes cross the $2m_{0}$ level, while additional contacts on the H side are limited by saturation. This qualitative pattern is consistent with the observed increase in $n_{M}(W)$ relative to $n_{H}(W)$ and the resulting dominance flip reported in the Dominance Flip Between H and M Groups subsection.

This mechanism is consistent with constrained-interaction views that separate opportunity (W) from routine-contact minimum ($m_{0}$) in the W–$m_{0}$ framework [17], and with previous work showing that short-time reshuffling and intercommunity bridges strongly influence invasion routes and timing in modular networks [10,11].

### Contribution of the Framework and Implications

Methodologically, this study combines 3 components in a single, interpretable framework. First, the W–$m_{0}$ setup separates a daily opportunity upper bound W from a routine-contact minimum $m_{0}$, and introduces $W_{random}$ as a separate control for random encounters (refer to Notation and Key Quantities subsection). Second, these quantities are implemented on a multilayer contact backbone with community structure and realistic venue types (household, school, workplace, distance-driven layers, and a random layer; refer to Model Overview, Data and Synthetic Population, Multilayer Potential Contact Network and H/M Classification, and Daily Contact Sampling Under the W–$m_{0}$ Framework subsections). Third, an extended SEIR model with vaccination and mild or severe branches is run on the daily graphs $\left\{G_{t}\right\}$, with contact-related parameters calibrated from an online survey in Tokyo and Kanagawa (refer to Survey-Based Calibration of Baseline Caps subsection). Within this setting, a small set of dominance-related quantities, $\left\{s_{i}(W),n_{i}(W),e_{i}(W),W^{\text{*}}\right\}$, summarizes which degree-based group (H or M) contributes most to transmission under each contact scenario. Thus, the framework retains structural richness while keeping the key indicators of “who leads spread” simple and comparable across settings.

Substantively, the results refine common intuitions about targeting high-contact individuals. Many models and narratives implicitly assume that high-contact individuals are always the main drivers of spread. In our multilayer, community-structured network, this holds when random-contact intensity is low, for $W_{random}\text{§amp;lt;}W_{random}^{\text{*}}$ the dominance score is larger for H. However, as random-contact intensity increases and $W_{random}$ passes the empirical threshold $W_{random}^{\text{*}}\approx 9.8$, the medium-contact group M becomes dominant. This shows that changes in random-contact opportunities can flip which part of the population contributes most to transmission, even when the overall opportunity bound W and the routine-contact minimum $m_{0}$ are fixed. This result complements previous bridge-focused and high-degree targeting narratives by showing that who occupies effective bridging positions can change when incidental encounters increase, even if per-contact transmissibility is unchanged.

These findings have direct implications for intervention priorities. When $W_{random}\text{§amp;lt;}W_{random}^{\text{*}}$, most effective transmissions originate from the H group. In this regime, measures that concentrate on H-type settings—such as large workplaces, dense households, and other high-degree environments—are expected to yield the largest marginal reductions in transmission (eg, focused screening or vaccination and testing outreach around identified H clusters). As $W_{random}$ approaches or exceeds $W_{random}^{\text{*}}$, random encounters play a larger role and the contribution of M increases. In this regime, reducing random-contact opportunities becomes important. In our framework, such measures aim to decrease the effective value of $W_{random}$ or of the gap $W\text{-}m_{0}$, for example, by limiting the size and frequency of large events, spreading flows over time through staggered commuting or opening hours, and reducing dense queues or long stays in crowded transfer spaces. Viewed through the dominance metric, these measures limit the formation of new intercommunity bridges and help prevent a shift from H-dominant to M-dominant spread.

### Limitations

Several limitations should be noted.

First, the synthetic population is restricted to a subsample of the Tokyo metropolitan area, and the layer-wise parameters were calibrated using 1 online survey in Tokyo and Kanagawa (refer to Survey-Based Calibration of Baseline Caps subsection), which is generally limited in terms of generalizability. This design may not represent typical daily behavior and may limit generalizability. The study is therefore not intended as a full reproduction of real-world contact patterns across settings. Instead, it is a scenario analysis focused on higher-activity conditions, where contacts are elevated and dominance shifts can be examined more clearly. Low-activity periods are outside the scope of this work.

Second, the quantitative value of the threshold $W_{random}^{\text{*}}$ depends on epidemiological parameters, such as infection probabilities and progression rates. Our structural conclusion—that increasing $W_{random}$ in a heterogeneous, multilayer network can flip dominance from H to M—relies on the presence of degree and layer heterogeneity together with an increasing $W_{random}$, rather than on a specific point estimate of any single parameter. We address uncertainty from randomness in daily contact sampling and transmission by reporting replicate runs under fixed parameter settings. However, these replicate runs do not replace a full sensitivity analysis over epidemiological assumptions (eg, infection rates, vaccine effects, and severity parameters).

Third, the operational definitions of the random-contact layer and of the H/M groups may differ across settings. In this study, H and M are defined by degree quantiles on the potential network, and the random layer aggregates several types of chance encounters; other implementations that respect the W–$m_{0}$ structure are possible.

Finally, our results are averages over a finite number of stochastic realizations. Even under the same model setting, the estimated value of $W_{random}^{\text{*}}$ varies across simulation runs due to randomness in daily contact sampling and transmission events. Importantly, while the point estimate of $W_{random}^{\text{*}}$ shifts across realizations, the qualitative tendency for an H→M dominance flip as $W_{random}$ increases remains robust.

### Conclusion

In this study, we modeled urban daily life as a multilayer contact network with a constrained, scale-free–like backbone and SEIR dynamics with vaccination. Individual contacts were governed by per-layer opportunity caps and per-layer routine-contact minima, together with a separate parameter for random encounters, and these quantities were calibrated using survey data from Tokyo and Kanagawa. On this backbone, we followed the epidemic on daily resampled networks and evaluated which degree-based group—H or M individuals—contributed most to transmission.

Within the range of calibrated settings, higher overall contact opportunities led to earlier and higher epidemic peaks and larger cumulative deaths, while lower opportunities slowed and prolonged spread. Beyond this expected pattern, our main finding is that changing random-contact intensity, even when overall contact opportunities and routine-contact minimum are held fixed, can flip which group dominates the spread. When random encounters are limited, most effective transmissions arise from the high-contact group. As random contact increases, saturation on the H side and the proliferation of intercommunity bridges through medium-contact individuals together shift the main contribution to the M group. In other words, who leads spread depends not only on baseline contact opportunities, but also on how many of those contacts are chance encounters that connect different communities. Replicate simulations further quantify stochastic uncertainty under fixed settings, showing that W* varies within a measurable range while the flip is consistently observed.

These results suggest that intervention priorities should reflect the level of random contact. When random encounters are relatively rare, focusing resources on high-contact settings—such as large workplaces and dense households—is expected to be most effective, because most transmission chains are rooted in these environments. When random encounters are frequent, reducing chance encounters and cross-community mixing becomes more important. In this framework, such measures can be interpreted as lowering the effective intensity of random contact or narrowing the within-layer gap between opportunity caps and routine-contact minima, for example, by limiting crowding and dwell time in shared spaces, staggering flows over time, and avoiding prolonged close contact with people outside one’s usual circles.

This work has several limitations and is one step in a longer line of research. The synthetic population and layer parameters are based on one metropolitan area and a COVID-19–like infection, and the classification into high- and medium-contact groups is only one possible way to summarize heterogeneity. Network dynamics are driven by daily resampling under fixed caps, without explicit behavioral feedback or seasonal change. Future studies should apply the same framework to other regions and time periods, investigate how the dominance flip and its threshold vary under different epidemiological and behavioral conditions, and combine additional data sources to refine the calibration of contact opportunities, routine-contact minimum, and random encounters. Even with these simplifications, the results of this study indicate that distinguishing between routine and random contacts, and tracking which group dominates spread, can provide a simple and interpretable guide for thinking about targeted control in multilayer urban networks.

## Abbreviations

H/M: high-contact and medium-contact
SEIR: Susceptible-Exposed-Infectious-Removed
